# Extracellular Vesicles in Acute Myeloid Leukemia: Opportunities and Obstacles for Therapeutic Advancement

**DOI:** 10.1002/jbt.70999

**Published:** 2026-07-20

**Authors:** Rongrong Hu, Zaker Ataullev, Luping Lou

**Affiliations:** ^1^ Yongkang Traditional Chinese Medicine Hospital Medical Community Xicheng Branch Jinhua Zhejiang China; ^2^ Natural Science Department University of Economics and Service Urgench Uzbekistan; ^3^ Department of Clinical Laboratory Shaoxing Central Hospital Shaoxing Zhejiang China

**Keywords:** acute myeloid leukemia, extracellular vesicles, hematological malignancies

## Abstract

Acute myeloid leukemia (AML) is a disease characterized by uncontrolled proliferation and blocked maturation of hematopoietic stem and progenitor cells. Despite progress in its clinical management, many patients continue to relapse or show limited response to standard therapies, emphasizing the need for novel, safe, and more effective targeted treatment approaches. In recent years, research has increasingly focused on uncovering the mechanisms responsible for treatment failure to optimize therapeutic strategies. Particular attention has been directed toward the role of extracellular vesicles (EVs) released by AML cells and the ways in which the tumor microenvironment (TME) adapts to promote leukemia progression and survival. Concurrently, advances in biotechnology have led to growing interest in using EVs as therapeutic tools in hematologic malignancies. EVs can function as carriers that induce apoptosis in cancer cells or suppress oncogene expression through their RNA cargo. Therefore, this review aims to synthesize and discuss recent discoveries on the roles and therapeutic potential of EVs in AML.

## Graphical abstract

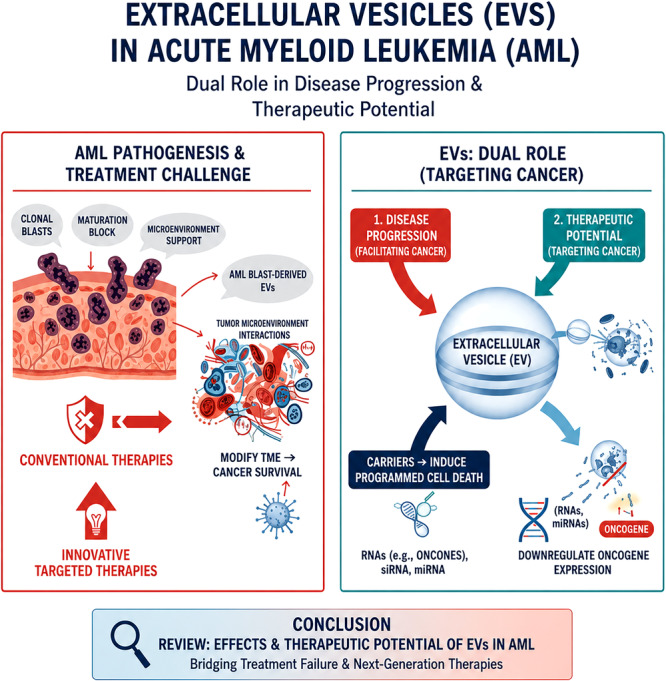

## 1 Introduction

Acute myeloid leukemia (AML) is a physiologically heterogeneous malignancy caused by clonally proliferating hematopoietic stem cells (HSC). AML is the most prevalent form of leukemia in adults, which accounts for only 1% of all cancer cases [1]. The majority of AML cases are diagnosed in individuals over the age of 60. Multiple risk factors, including myeloproliferative disorders, genetic predispositions, environmental exposures, and prior chemotherapy or radiation, are associated with AML. In recent years, understanding the molecular biology of AML has significantly improved its characterization and introduced novel therapeutic approaches [2].

Extracellular vesicles (EVs), including microvesicles (MVs), exosomes, and exosome‐like vesicles (ELVs), hold significant promise among the emerging innovative approaches in cancer immunotherapy. EVs play a crucial role in cell‐to‐cell communication by transporting diverse types of RNAs such as messenger RNAs (mRNAs), ribosomal RNAs (rRNAs), transfer RNAs (tRNAs), microRNAs (miRNAs), long non‐coding RNAs (LncRNAs), circular RNAs (circRNA), proteins, and lipids from the donor cell to the target cell [3, 4, 5].

Consequently, EVs serve as a crucial mechanism for intercellular cross‐talk. Within this framework, cancer cells can use EVs to alter the tumor microenvironment (TME) and create a tumorigenic niche, resulting in metastasis and tumor development [6]. EVs may also affect immune evasion by creating an immunosuppressive milieu, leading to enhanced chemoresistance and poor prognosis [7, 8]. On the other hand, EVs have the potential to become indispensable tools in the treatment of hematological malignancies by either acting as carriers that induce the programmed cell death of malignant cells or by downregulating the expression of oncogenes [9]. This review discusses recent findings on the impact and therapeutic potential of EVs in AML. Although the research on EVs is still in its early stages, remarkable advancements in biotechnology offer promising possibilities for reshaping these vesicles and improving the treatment of hematological cancers.

## 2 Literature Search Methodology

A comprehensive literature search of Web of Science, PubMed, and Scopus databases covering January 2000 through December 2024 was conducted using keywords “Extracellular vesicles,” “exosomes,” “EV,” “microvesicles,” “acute myeloid leukemia,” and “AML.” Only English peer‐reviewed papers with relevant outcomes were considered. A standardized data extraction Excel form was subsequently utilized to compile pertinent information from the included studies, encompassing the author's name, publication year, study type, sample size, intervention type, dosage, and outcomes. Additionally, we endeavored to independently assess each paper and elucidate the discrepancies in the results.

### 2.1 EV Biogenesis

EVs, a diverse collection of membrane‐structured vesicles released by almost all living cells, were initially overlooked due to the long‐held belief that they constituted cell waste. Consequently, the distinction between EVs and non‐EV particles remained elusive. However, in the past two decades, the study of EVs has experienced exponential growth, fueled by a resurgence of interest in the subject in the early 2000s, after decades of limited recognition of these structures [10]. In 2011, the term “EVs” was coined to encompass a diverse range of membrane vesicles that are excreted by cells [11]. In addition to transporting proteins, nucleic acids, and lipids from donor cells to recipient cells, EVs also contain a variety of bioactive molecules [12]. EVs have a significant role in a variety of pathophysiological events and act as crucial mediators of cellular and molecular crosstalks [13].

Researchers define EVs based on their physical characteristics, such as size and density, or their biological structure or source. However, individual markers of EV subtypes require further investigation. The majority of EVs found in biological fluids are small, ranging from 50 to 150 nm in diameter. Medium‐sized EVs (200−800 nm) are less common, while large EVs (≥ 1000 nm) are extremely rare (Table 1). Previous research has often categorized EVs into three main groups: exosomes, MVs, and apoptotic bodies [3]. Endocytosis and multivesicular body (MVB) generation are some of the complicated mechanisms that create exosomes, the smallest subpopulation of EVs with a size range of 30−200 nm [14]. MVs, which have a diameter of 100–1000 nm, are produced by the direct outward branching of plasma membranes [15], and apoptotic bodies are > 1000 nm vesicles released during apoptosis [16]. Based on the standards provided by the Minimal Information for Studies of Extracellular Vesicles 2018 (MISEV2018), exosomes, MVs, and apoptotic bodies are all referred to as “EVs” in this study [17].

**Table 1:** EV categories based on diameter.

Category	Small	Medium	Large
Diameter	50–150 nm	200–800 nm	≥ 1000 nm
Biogenesis	Endosomal (exosomes) but some small EVs can be derived from the plasma membrane (ectosomes)	Plasma membrane‐derived ectosomes	Plasma membrane‐derived ectosomes (some of which may carry endosomal small EVs)
Members	Exosomes, small ectosomes, ciliary ectosomes, arrestin domain‐containing protein 1‐mediated microvesicles	Microvesicles, FDC‐derived iccosomes, T cell microvilli particles, elongated neutrophil‐derived structures, secreted midbody remnants	Apoptotic bodies, large oncosomes, beaded apoptopodia, migrasomes, exophers, MVB‐like EV clusters, secretory autophagosomes, cytoplasts

Abbreviations: EVs, extracellular vesicles; FDC, follicular dendritic cell; MVB, multivesicular body.

**Figure 1:**
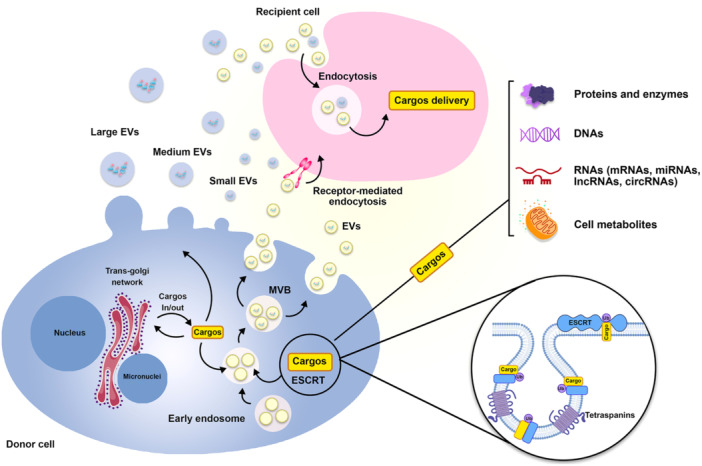
ESCRT‐dependent pathway of EV biogenesis. There are several mechanisms involved in the production of extracellular vesicles (EVs). These processes include the synthesis of early endosomes, multivesicular body (MVB) formation, and the fusion of MVBs with plasma membranes. The ESCRT‐dependent biogenesis of exosomes initiates with the assembly of ESCRT complexes on ubiquitin (Ub)‐bound cargo. Once ESCRTs bind to the Ub‐cargo, the mammalian tumor susceptibility gene 101 (TSG101) facilitates the internalization of the cargo within the exosome. Following endocytosis, membrane fusion, and receptor−ligand contact, EVs deliver cargo such as proteins, nucleic acids, and lipids to recipient cells.

To date, several separation techniques have been developed to ensure the purity, integrity, and yield separation of EVs, each with several advantages and disadvantages. These methods encompass ultracentrifugation, density gradient centrifugation, size‐exclusion chromatography, and precipitation [18, 19]. Additionally, techniques such as RNASeq and qPCR have been employed for cargo profiling of EVs. Nevertheless, the precise mechanisms underlying cargo sorting into EVs remain elusive. One of the widely recognized theories proposed to elucidate this phenomenon is the ubiquitin (Ub)‐dependent loading of proteins into MVBs through the endosomal sorting complexes required for the transport (ESCRT) pathway [20]. The ESCRT‐dependent biogenesis of exosomes initiates with the assembly of ESCRT complexes on Ub‐bound cargo (Figure 1). Once ESCRTs bind to the Ub‐cargo, the mammalian tumor susceptibility gene 101 (TSG101) facilitates the internalization of the cargo within the exosome. This intricate process is meticulously regulated by a network of key proteins, including members of the tetraspanin protein family (CD81, CD63, and CD9), proteins involved in membrane fusion (Annexin A1‐2, Annexin A7, Rab7, and Rab5), proteins responsible for the synthesis of MVBs, chaperone proteins, and phospholipases [21]. There are also a lot of unique proteins in EVs produced from various donor cells, and those proteins could dictate what the EVs perform biologically. Nucleic acids are another critical component of EVs. EVs are increasingly demonstrated to regulate the biological activities of target cells by delivering mRNAs, rRNAs, tRNAs, miRNAs, circRNAs, lncRNAs, mitochondrial DNA, and genomic DNA [22, 23].

### 2.2 The Significance of EVs in Cancer

**Figure 2:**
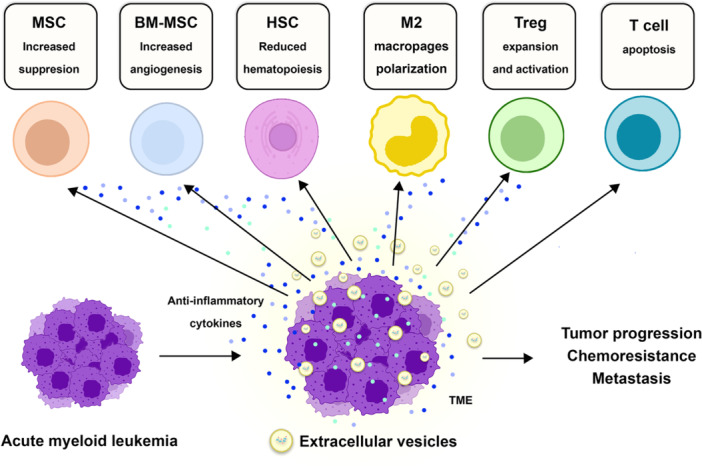
Schematic illustration of the pathogenic role of EVs in AML progression. Extracellular vesicles (EVs) mediated crosstalk between acute myeloid leukemia (AML) blasts and effector cells in the tumor microenvironment (TME). AML EVs induce Treg expansion and apoptosis in the effector T cells. AML EVs also facilitate anti‐inflammatory M2 polarization of macrophages. Hematopoietic stem cells (HSCs) colony formation is negatively impacted by AML EVs, resulting in hematopoiesis disruption. Anti‐inflammatory cytokine production is also increased in the TME, resulting in tumor progression, metastasis, and immune evasion. AML, acute myeloid leukemia; BM‐MSC, bone marrow mesenchymal stromal cell; EVs, extracellular vesicles; HSC, hematopoietic stem cell; TME, tumor microenvironment; Treg, regulatory T cells.

The most significant characteristics of human malignancies, including angiogenesis, immune evasion, resistance, metastasis, and cancer development, are all influenced by EVs (Figure 2). Hypoxia and acidic extracellular milieu, major characteristics of TME, are highly associated with the release of tumor EVs [24]. Hypoxia triggers the secretion of EVs, which subsequently facilitate the migration of microvascular endothelial cells and the process of angiogenesis [25, 26]. Low pH also triggers an enhanced release of EVs from tumor cells, resulting in epithelial−mesenchymal transition (EMT), a process whereby tumor cells diminish E‐cadherin expression and develop metastasis [27]. Cytokines and growth factors such as hepatocyte growth factor (HGF), epidermal growth factor (EGF), and transforming growth factor beta (TGF‐β) may also transfer signals that activate the EMT process [28, 29]. As a crucial stage in metastasis, tumor‐derived EVs possess proteases and the ability to destroy the extracellular matrix (ECM). EVs may also cause endothelial cell growth and migration, resulting in abnormal angiogenesis. This triggers EC proliferation, which causes the secretion of matrix metalloproteases (MMPs), further breaking down the ECM and promoting tumor development. The release of EVs that sequester anti‐cancer medications is another escape mechanism by which tumor cells develop multidrug resistance (MDR) [30]. The ATP‐binding cassette (ABC) transporters, which are newly identified in EVs, seem to have an important role in this process. EVs produced from tumors may also regulate the activation of lymphocytes by providing immune‐modulatory molecules and promoting immune evasion [31]. EVs can also help cancer cells hide tumor‐associated antigens (TAAs), propagating immune escape and enhancing tumor progression [32].

### 2.3 EVs in AML Pathogenesis

AML is a heterogeneous and acute hematological malignancy that involves the abnormal proliferation of myeloblasts or progranulocytes. The existence of circulating blasts is evaluated in a bone marrow (BM) biopsy or peripheral blood sample, which establishes the diagnosis. The diagnosis can be confirmed by flow cytometry with the existence of more than 20% immature myeloid cells [33, 34]. Leukemic stem cells (LSCs) and their cancerous compartments, mesenchymal stem cells (MSCs), and HSCs comprise the BM microenvironment. The presence of blast‐derived EVs in AML patients has been reported, and this finding is correlated with disease severity and recurrence, suggesting that EVs play a significant role in AML pathophysiology [35]. In order to shape stromal and hematopoietic progenitor cells and to favor regulatory TME, AML cells secrete an excessive amount of EVs [36]. This is supported by the work of Huan et al., who demonstrated that BM stromal cells were able to regulate proliferation signaling and increase vascular endothelial growth factor (VEGF) levels through the adoptive transfer of EVs derived from AML cells [37]. In addition, the examination of EVs derived from HL‐60 AML cell lines revealed that BM stromal cells were able to capture these EVs, a phenomenon that consequently changed the pattern of growth factor secretion [38]. On the other hand, by changing the niche stroma, EVs produced from AML indirectly inhibit the activity of HSCs. The inhibition of stem cells by AML EVs, which produce chemokines like stromal cell‐derived factor 1 (SDF‐1), and the reduction of hematopoiesis essential transcription factors like CCAAT enhancer binding protein β (CEBPB) and c‐terminal domain‐containing protein (c‐Myb), allows HSCs to migrate from the BM and ultimately leads to the inhibition of the normal formation of blood cellular components [39]. Besides, human‐to‐mouse tumor‐derived EVs inhibited osteoblasts, cells that form bone tissue, in stromal progenitor cells and prevented bone development in vivo [40]. Studies have also shown that AML EVs decrease the expression of several molecules involved in normal hematopoiesis, including collagen type I alpha chain (COL1A1), osteocalcin, SDF‐1, insulin‐like growth factor (IGF), and interleukin (IL)‐7 [41]. The EVs originating from AML contain different miRNA patterns compared to their normal counterparts, including miR‐375, miR‐223, miR‐191, miR‐155, miR‐150, miR‐125b, miR‐99b, miR‐9, and let‐7a [42, 43]. EVs generated from AML‐derived cells had a higher concentration of miR‐7977, a suppressor of the major cellular poly(rC)‐binding protein 1 (PCBP1), than CD34^+^ HSC‐derived EVs [44]. The significant effects of these miRNAs extend beyond the inhibition of hematopoiesis since their correlation with tumor development, recurrence, and prognosis has also been investigated. Studies have shown a strong link between miR‐125b expression and AML's poor prognosis, suggesting that EVs derived from AML play a role in literally every stage of AM progression [45].

The establishment of leukemic niches is essential for the migration of immature myeloid blasts to secondary organs. Leukemic cells in circulation may modulate the TME through the development of malignant niches. AML cells release EVs that may modify the immune system and enhance chemoresistance, hence promoting tumor progression [46, 47]. AML‐derived EVs contain a range of TAAs like CD44, IL‐3Rα, chemokine (C−C motif) ligand 1 (CCL1), and T cell‐activated increased late expression (TACTILE), as well as blast indicators like CD34, CD33, and CD117. However, a notable pathological characteristic of these EVs is a higher level of immunosuppressive factors like immune checkpoints programmed death‐1 (PD‐1), programmed death‐ligand 1 (PDL‐1), major histocompatibility complex (MHC) class I chain‐related proteins A and B (MICA and MICB), adenosine receptors CD39 and CD73, Fas ligand (FasL), and TGF‐β [48, 49, 50]. It was discovered that EVs that contained TGF‐β and natural killer group 2D ligands (NKG2DL) were able to inhibit the cytotoxicity of natural killer (NK) cells, one of the main effector cells in anti‐cancer immunity [51]. EVs may also be captured by dendritic cells (DCs) in TME, resulting in reduced interferon (IFN)‐γ secretion [52, 53]. According to Szczepanski et al., chemotherapy has the potential to decrease the amount of TGF‐β in EVs from AML patients, suggesting that this cytokine might likely play a role in the development of AML [54]. So, since TGF‐β is a key factor for regulatory T cells (Tregs), AML EVs also affect immune regulation by encouraging the development of these populations [55]. Wieckowski et al. reported a reduction in signaling molecules, including CD3 and tyrosine‐protein kinase Janus kinase 3 (JAK3), in stimulated T cells when exposed to AML EVs, along with reduced phosphorylation of signal transducer and activator of transcription 5 (STAT5) in effector cytotoxic T cells (CTLs) and lower levels of T helper (Th) cells [56]. EVs produced from AML cell lines had a significant impact on CTL growth and enhanced Treg proliferation. Hong et al. demonstrated that these EVs may trigger apoptosis in CD8^+^ CTLs [57, 58].

AML tumor cells also cause hypoxia in the BM microenvironment. This process encourages the production of EVs from tumors or MSCs, which regulates hypoxia‐inducible factor 1 subunit alpha (HIF‐1α), increases the expression of TGF‐β, and accelerates the development of AML [59]. AML cells are using a variety of strategies to promote chemoresistance. EVs derived from AML patients express VEGF, which induces endothelium remodeling and promotes chemoresistance [60]. AML cells regulate the expression of genes linked to apoptosis in chemosensitive blasts. In addition, B‐cell lymphoma‐extra‐large (Bcl‐xL) and B‐cell lymphoma 2 (BCL‐2) are anti‐apoptotic molecules that AML EVs transmit, which makes cancer cells less responsive to chemotherapy [61]. The autocrine mechanism by which AML EVs induce chemoresistance was recently elucidated by Hong et al. Because AML blasts contain the enzyme 3‐hydroxy‐3‐methyl‐glutaryl coenzyme A reductase on their EVs, the cancer cells develop chemoresistance. This resistance is caused by a chain reaction that begins with an increase in cholesterol synthesis and ends with EVs shedding into the extracellular space [62]. Apoptosis‐resistant AML blasts may similarly become resistant to chemotherapy by transferring EVs, which modify the gene regulatory axis and diminish reactive oxygen species (ROS) production [63]. Bouvy et al. showed that a resistant profile may be transmitted by AML EVs from chemo‐resistant to chemo‐sensitive cells [64]. Recent investigations indicated that the progression of leukemia is significantly postponed by inhibiting the production of AML EVs by targeting Rab27a [65]. Vacuolar protein sorting 33b (VPS33B) is another crucial molecule in the creation of AML EVs; Deletion of VPS33B leads to a reduction in EV production and subsequently delays AML development [66]. Therefore, treatment using vitamin A derivatives such as all‐trans‐retinoic acid (ATRA) may influence AML EVs by changing their properties. ATRA reduces the pro‐coagulant properties of NB4 cells‐derived EVs in endothelial cells [67]. In summary, leukemia‐derived EVs enhance tumor growth while inhibiting normal hematopoiesis; however, EVs from normal BM cells have distinct effects, which may be due to their different cargos. Consequently, the inhibition of AML EVs, along with their reshaping to boost anti‐tumor immunity, appears to be a compelling strategy in AML therapy.

### 2.4 EVs as Therapeutic: Preclinical Evidence

The last century has seen several initiatives aimed at reducing cancer‐related deaths via the use of various approaches, including cytotoxic medications, small‐molecule inhibitors (SMIs), and targeted antibodies [68]. However, there are still limitations in cancer therapy and gaps in introducing safe and efficient approaches. Chemotherapy is one of the primary methods of treating AML; nevertheless, many patients develop resistance to these medications, which results in treatment failure and a shorter life expectancy [69]. In recent years, researchers have employed several novel strategies with varying degrees of success (Table 2). Recent advancements in AML management include mutation‐specific targeted treatments such as midostaurin, stem‐cell transplantation (SCT), isocitrate dehydrogenase 1 (IDH1) inhibitors, IDH2 inhibitors, immune checkpoint blockers (ICBs), bi‐specific T‐cell engagers (BiTEs), chimeric antigen receptor (CAR) T‐cells, and CAR‐cytokine‐induced killer cells (CIKs) [70, 71, 72]. However, there is currently no recommended first‐line treatment with appropriate efficacy in most patients. Recently, reshaping EVs to treat cancer cells has introduced new optimism into the field. As mentioned before, EVs are nanovesicles that have great promise for the treatment of AML and other malignancies because of their distinctive features.

**Table 2:** Summary of EV‐based studies in acute myeloid leukemia.

Study ID	Model	EV Source	Cargo/target	Signaling pathway	Main result	Limitations
Zhang 2020	In vitro (THP‐1 AML cells)	Human BM‐MSC‐derived exosomes	miR‐222‐3p → IRF2/INPP4B	IRF2/INPP4B signaling axis	Suppressed proliferation and induced apoptosis in AML cells	In vitro only; single cell line; no in vivo validation
De Luca 2016	In vitro + NSG mouse model	BM‐MSC‐derived EVs	miR‐27b/MPL, miR‐181/EGR2, miR‐21/ANXA1	CXCR4‐mediated homing pathways	Enhanced survival, reduced differentiation, and increased BM homing	Focused on HSCs; indirect AML therapeutic relevance
Jalilivand 2024	In vitro (HL‐60 AML cells)	BM‐MSC exosomes	JAK2/STAT3/STAT5	JAK/STAT signaling pathway	Suppressed JAK/STAT signaling in AML cells	In vitro only; limited cargo characterization
Wen 2023	In vitro + humanized AML mouse model	Engineered MSC‐derived exosomes	miR‐34c‐5p → LSCs	miR‐34c‐5p downstream apoptotic pathways	Selective eradication of LSCs; suppressed AML progression	Preclinical stage; delivery scalability not addressed
Jiang 2022	In vitro AML cells	BMSC‐derived exosomes	miR‐7‐5p → OSBPL11	PI3K/AKT/mTOR axis inhibition	Reduced proliferation; increased apoptosis	No in vivo validation; AML heterogeneity not addressed
Yang 2024	In vitro AML cells	Reovirus‐infected UC‐MSC exosomes	Oncolytic reovirus	Clathrin‐mediated endocytosis (CME)	Delivered virus; induced AML cytotoxicity	Viral safety concerns; limited in vivo data
Khatib 2024	In vitro (KG‐1 AML cells)	NK and LAK cell‐derived exosomes	Cytotoxic granules (perforin/granzymes)	Apoptotic cytotoxic pathways	LAK‐Exo induced higher apoptosis than NK‐Exo	In vitro only; single AML line
Namburi 2021	In vitro + patient plasma samples	AML patient‐derived exosomes	CD26 (DPP4)	DPP4‐mediated myelosuppressive signaling	Suppressed HSC colony formation; reversed by DPP4 inhibition	Mechanistic focus; not a therapeutic modification study
Zhao 2019	In vitro (CD34^+^ HSCs)	AML‐derived exosomes	miR‐4532 → DKK1	LDOC1‐dependent STAT3 signaling pathway	Impaired normal hematopoiesis	No therapeutic intervention tested
Du 2023	In vitro + mouse AML model	AML‐derived exosomes	circ001264 siRNA + anti‐PD‐L1	RAF1/STAT3 axis; PD‐L1 immune checkpoint	Reduced M2 polarization; decreased tumor burden	Combination strategy complexity; translational uncertainty
Bi 2021	In vitro (pediatric AML)	AML‐derived exosomes	circ0004136 → miR‐570‐3p/TSPAN3	miR‐570‐3p/TSPAN3 regulatory axis	Knockdown impaired AML growth; increased apoptosis	Primarily in vitro; pediatric‐specific focus
Su 2024	In vitro + transgenic mouse model	circBMI1‐modified exosomes	circBMI1 → miR‐338‐5p/ID4	miR‐338‐5p/ID4 regulatory pathway	Suppressed AML progression; increased drug sensitivity	Early preclinical stage; long‐term safety unknown

Abbreviations: AKT/PKB, protein kinase B; AML, acute myeloid leukemia; BM, bone marrow; BMSC, bone marrow mesenchymal stem cell; CFU, colony‐forming unit; CME, clathrin‐mediated endocytosis; CXCR4, C−X−C chemokine receptor type 4; DPP4, dipeptidyl peptidase 4; EV, extracellular vesicle; HSC, hematopoietic stem cell; JAK, Janus kinase; LAK, lymphokine‐activated killer; LSC, leukemia stem cell; MSC, mesenchymal stem cell; mTOR, mammalian target of rapamycin; NK, natural killer; NSG, NOD SCID gamma; PD‐L1, programmed death‐ligand 1; PI3K, phosphoinositide 3‐kinase; RAF1, rapidly accelerated fibrosarcoma 1; STAT, signal transducer and activator of transcription; UCB, umbilical cord blood.

Zhang et al. investigated the role of BM‐MSCs‐Exo, which are exosomes produced from human BM‐MSCs, in modulating proliferation and apoptosis. They used the cell counting kit‐8 (CCK‐8) and flow cytometry to analyze cellular proliferation and apoptosis. Additionally, they utilized quantitative real‐time polymerase chain reaction and immunoblot analysis to examine the expression of miR‐222‐3p. According to the findings, BM‐MSCs are responsible for the transfer of miR‐222‐3p to THP‐1 cells via exosomes, resulting in the inhibition of cell proliferation and inducing apoptosis [73]. De Luca et al. demonstrated that umbilical cord blood CD34+ stem cells (UCB‐CD34^+^) exposed to BM‐MSC‐derived EVs significantly altered several biological functions. They identified approximately 100 downregulated genes among those targeted by EV‐derived miRNAs, including miR‐27b/MPL, miR‐181/EGR2, and miR‐21/ANXA1. This suggests that EV content was capable of modifying the gene expression profile of receiving cells. Consequently, UCB‐CD34^+^ cells became more viable and less differentiated, and exhibited an increase in C−X−C chemokine receptor type 4 (CXCR‐4) expression. This was accompanied by an increase in in vivo migration from peripheral blood to the BM niche in NOD SCID gamma (NSG) mice [74]. Jalilivand et al. also demonstrated a substantial reduction in the expression of signal transducer and activator of transcription 3 (STAT3), STAT5, and Janus kinase 2 (JAK2) in HL‐60 cells that were treated with BM‐MSC exosomes [75]. Wen et al. concentrated on eliminating LSCs in individuals with AML. The objective of the study was to mitigate the elevated risk of disease recurrence posed by these patients' ability to eliminate LSCs. MiR‐34c‐5p was employed in this investigation. Engineered MSC‐derived exosomes were used to efficiently transport miR‐34c‐5p to LSCs. Engineered exosomes containing miR‐34c‐5p were shown to preferentially remove LSCs and suppress AML development in a humanized AML mouse model. Their study effectively established an efficient delivery mechanism, providing new insights into potential therapeutics for administering miRNA or other drugs to LSCs [76].

Jiang et al. administered exosomes enriched with miR‐7‐5p from MSCs to AML cells to investigate the control of cell−cell interaction. The research indicated a reduction in the expression of miR‐7‐5p in both AML patients and AML cells. Reduced cell proliferation and higher apoptosis were seen in AML cells after treatment with miRNA‐enriched EVs. MiR‐7‐5p‐enriched EVs originating from BMSCs inhibited the phosphorylation of the phosphoinositide 3‐kinases (PI3Ks)/protein kinase B (PKB)/mammalian target of rapamycin (mTOR) axis, resulting in a higher level of the oxysterol binding protein‐like 11 (OSBPL11) gene. miR‐7‐5p overexpression inhibits cell proliferation and promotes apoptosis, suggesting that EVs originating from BMSCs may improve the therapeutic efficacy in AML [77]. Yang et al. also demonstrated that exosomes from reovirus‐infected umbilical cord‐derived MSCs (MSCREO‐EXOs) exhibited cytotoxicity against AML cells and facilitated the transmission of reovirus to tumor cells primarily via clathrin‐mediated endocytosis (CME). They also showed that MSC‐EXOs can be used to induce anti‐tumor effects on AML cells by delivering oncolytic viruses [78].

Khatib et al. examined the anti‐tumor efficacy of NK cell‐derived exosomes (NK‐Exo) and activated NK cell‐derived exosomes (LAK‐Exo) against the KG‐1 AML cell line. The findings demonstrated the efficacy of NK‐Exo against the KG‐1 cell line in vitro; however, the killing capacity of LAK‐Exo on target cells was much greater than that of NK‐Exo. Additionally, after 48 h of coculture with NK‐Exo and LAK‐Exo, respectively, the overall apoptosis seen in AML cells was 34.56% and 51.6% [79]. The regulation of hematopoiesis has been associated with CD26, a serine protease, as demonstrated by Namburi et al. CD26 was transported by EVs derived from leukemia cell lines or plasma from AML patients. It was shown that the CD26 activation in AML EVs, which inhibited the development of HSC colonies, was much greater than the CD26 activity observed in the EVs of normal individuals. The exosome‐mediated myelosuppression was reversed by pharmacologically inhibiting DPP4 in AML exosomes, resulting in an improvement in cell count and patient recovery [80]. Zhao et al. identified miR‐4532 in AML‐derived exosomes as a suppressor of normal hematopoiesis. AML exosomes deliver miR‐4532 into CD34^+^ HSCs, which reduces colony‐forming unit (CFU) output and increases expression of DKK1, a Wnt inhibitor that blocks HSC growth. Conversely, blocking miR‐4532 in AML exosomes restored CFUs and lowered DKK1 expression. These results show that exosomal miRNAs from leukemia cells may impair HSC function [81].

For anticancer therapy in vitro and in vivo mouse models, Du et al. used an anti‐ programmed death‐ligand 1 (PD‐L1) antibody in conjunction with exosomal circ001264 small interfering RNA (siRNA). Individuals with AML had an abnormally high expression of circ001264, and this upregulation was associated with a bad prognosis for patients. The exosomal circ001264, which was produced by AML cells, induced M2 macrophage differentiation by regulating serine‐threonine kinase (RAF1) transcription and activating the STAT3 axis. Through the secretion of PD‐L1, M2 macrophages have the ability to trigger PD‐L1 upregulation. The anti‐PD‐L1 medication was chosen for the purpose of avoiding immunosuppression, which was successful in producing the therapeutic impact against AML. In vivo, combining exosomal circ001264 siRNA with anti‐PD‐L1 significantly reduced leukemia tumor burden [82, 83]. Moreover, Bi et al. demonstrated that circ0004136 was upregulated in the serum and leukemia cells. The suppression of circ0004136 by exosomes impaired the survival, growth, and immigration of AML cells while enhancing apoptosis. Furthermore, circ_0004136 operated as a reservoir for miR‐570‐3p, whereas tetraspanin 3 (TSPAN3) served as a downstream target of miR‐370‐3p in AML. Through the downregulation of miR‐570‐3p, the inhibition of circ0004136 was reversed. Additionally, the higher levels of miR‐570‐3p inhibited the AML progression by decreasing TSPAN3 transcription [84]. Mice with circRNA‐polycomb ring finger proto‐oncogene (BMI1) upregulation exhibited decreased white blood cell counts, which indicated a less severe AML, as demonstrated by experiments conducted on circBMI1 transgenic models. *Su* et al. demonstrated that circBMI1 impeded AML growth by interacting with miR‐338‐5p, hence influencing the levels of inhibitor of DNA‐binding protein 4 (ID4). Exosomes isolated from circBMI1‐HL‐60 cells demonstrated tumor‐suppressing properties, including a reduction in HL‐60 proliferation, an increase in drug sensitivity, and the promotion of apoptosis [85].

In addition to the aforementioned signaling mediators, there are other axes that could also become targets for future therapies in AML. CCAAT/enhancer‐binding protein‐α (CEBPA) is a key transcription factor for myeloid cell differentiation and is required for normal neutrophil development and AML onset. Loss of CEBPA disrupts myelopoiesis and contributes to leukemogenesis. Notably, CEBPA has been identified as a target of miR‐125b, linking miRNA dysregulation to impaired CEBPA function in AML [86]. Core‐binding factor subunit β (CBFB) is also a target for miR‐125b. Bousquet et al. demonstrated that miR‐125b directly targets CBFB [87]. Overexpression of miR‐125b in normal hematopoietic stem and progenitor cells (HSPCs) in vitro and in vivo drives leukemic transformation [88]. Thus, CBFB downregulation by miR‐125b is linked to malignant transformation of HSPCs. The Myc family transcription factors are central to HSC maintenance. Laurenti et al. showed that c‐Myc activity controls HSC proliferation, differentiation, and survival. In particular, Myc activity is required for the proper balance between HSCP self‐renewal and differentiation [89]. GATA2 is a zinc‐finger factor critical for early hematopoiesis. It is co‐expressed with GATA1 and governs the development of erythroid and megakaryocyte lineages [90]. In AML, aberrant GATA2 activity may drive a pro‐inflammatory loop. Katsumura et al. showed that high GATA2 expression correlates with elevated C−X−C motif chemokine ligand 2 (CXCL2)/IL1B and predicts poor prognosis in AML [91]. CCL3 (MIP‐1α) is another inflammatory chemokine produced by AML blasts that remodels the BM niche. MIP‐1α/CCL3 axis can induce multiple processes that support the dominant proliferation of leukemia cells, suggesting that CCL3/MIP‐1α may act as an AML‐derived growth factor [92, 93].

### 2.5 Clinical Translation and Current Limitations

EV‐based treatments are in early development and show fewer adverse effects compared to cell therapies. Most research has been preclinical, involving in vitro and in vivo models, and few have reached clinical trials. This leaves unanswered questions about how EVs interact with the human microenvironment.

Translating EV research into AML therapies presents several challenges in the context of mass production and clinical application, encompassing concerns related to preparation, storage, and quality control. To advance the development of EVs for clinical applications, it is crucial to identify effective manufacturing techniques under Good Manufacturing Practice (GMP) conditions using clinical‐grade and high‐quality reagents. Presently, there are no established GMP standards for EVs in AML. However, a robust and scalable process encompassing cell expansion, EV harvesting, purification, and rigorous quality control is essential for future clinical translation. For instance, Humbert et al. reported a large‐scale GMP‐compliant strategy for the production of an EV‐enriched secretome derived from cardiovascular progenitor cells (CPC). This process encompasses the vesiculation of CPC, purification, and concentration of the product, as well as sterilizing filtration. This process successfully passed quality control tests and was approved for a Phase I trial for the treatment of heart failure. Results demonstrated safety and biological activity, and no signs of toxicity or malignancy development were observed in vivo [94].

Another significant issue is product heterogeneity and potency. Batch‐to‐batch variability in EV preparations can be high due to cell source differences and culture conditions. Efforts to address these issues include engineering EVs, preconditioning techniques, and the development of EV‐mimetic nanovesicles [95, 96]. Besides, applying novel approaches, such as well‐characterized cell banks or synthetic mimetics, may address some concerns about the standardization. Potency assays and cargo‐profiling are also required before clinical use. While EVs are generally well tolerated, they can carry immunostimulatory molecules that might provoke immune responses [97]. Therefore, any clinical EV product must be screened for immunogenicity and cleared of pathogenic agents.

## 3 Conclusion and Future Perspective

Recent years have witnessed a surge in studies exploring the active utilization of EVs as carriers capable of inducing apoptosis or repressing the expression of specific genes, including reshaping EVs to manipulate the expression of certain oncogenes using diverse types of RNAs. Moreover, EVs can selectively target malignant cells using surface protein molecules, which allows the delivery of targeted chemotherapy drugs while safeguarding the surrounding tissues from adverse effects. The in vivo and in vitro outcomes of these studies have been promising and encouraging in AML. Despite the potential therapeutic applications of EVs in AML, current research encounters several challenges in scalable GMP manufacturing, batch variability, biodistribution, immunogenicity, and off‐target effects.

To date, no clinical trials of EV‐based therapies in AML have been completed. EV trials are being pursued in other fields, but AML‐specific studies have yet to be initiated. This gap underscores the nascent state of EV therapeutics in leukemia. Furthermore, several methodological limitations impact the clinical translation of EV studies discussed in this review. Initially, it is important to note that studies on EVs vary significantly. Many studies utilize in vitro models, such as cell lines or ex vivo‐treated cells. Some studies employ murine AML models, while a limited number involve patient samples. In vitro findings, such as those obtained from cell lines like HL‐60 or U937, provide valuable insights but may not fully replicate the complexities of human AML biology. In vivo AML models, such as xenografts or syngeneic mice, offer greater predictive value but still lack the genetic diversity observed in patient disease.

Furthermore, the isolation and characterization methods employed in studies differ. Common approaches include differential ultracentrifugation, size‐exclusion chromatography, and commercial precipitation kits. These methods exhibit variations in yield, purity, and the co‐isolation of non‐EV materials, making it challenging to compare results across studies. Quantification and characterization methods also exhibit inconsistencies. Some studies report EV number using nanoparticle tracking, while others employ protein content or electron microscopy. ISEV's MISEV2018 guidelines recommend a quantitative description of both EV source and EV preparation, as well as analysis of multiple EV markers. However, few AML studies fully adhere to these recommendations, and many lack characterization of non‐EV co‐isolates. Additionally, since EV mediators exert their effects depending on the neoplastic context, any therapeutic approaches could yield different results in HSPC differentiation and related functions, contingent upon the specific disease.

In summary, while preclinical data are promising, more homogeneous studies and clinical trials are needed to gain deeper insights into the biological characteristics of EVs, which present an opportunity to improve their production methods, enhance their nanomedicine carrier functions, and increase their safety and overall effectiveness for therapeutic applications in AML.

## Author Contributions

This research was conducted in collaboration with all authors. Z.A. and L.L. were responsible for idea design, data collection, and literature selection. R.H. contributed to the final approval and manuscript writing.

## Funding

The authors have nothing to report.

## Ethics Statement

The authors have nothing to report.

## Consent

The authors have nothing to report.

## Conflicts of Interest

The authors declare no conflicts of interest.

## Data Availability

Data sharing is not applicable to this article, as no data sets were generated or analyzed during the current study.
